# Predictors of the amount of intake of Ready‐To‐Use‐Therapeutic foods among children in outpatient therapeutic programs in Nairobi, Kenya

**DOI:** 10.1002/fsn3.2745

**Published:** 2022-01-18

**Authors:** Sophie Ochola, Irene A. Ogada, Colleta A. Odera

**Affiliations:** ^1^ 107864 Department of Food, Nutrition and Dietetics Kenyatta University Nairobi Kenya; ^2^ Department of Applied Human Nutrition Mount Saint University Halifax Nova Scotia Canada

**Keywords:** adequate intake of Ready‐to‐Use Therapeutic food (RUTF), children 6–23 months of age, determinants, outpatient therapy program, predictors, severe acute malnutrition

## Abstract

Ready‐to‐use Therapeutic Food (RUTF) therapy is a standard protocol for treating children with severe acute malnutrition (SAM) admitted in Out‐Patient Therapeutic Programmes (OTP). The amount of RUTF to be consumed by a child is based on weight (200 kcal/kg body weight/day) as stipulated in the Kenya Integrated Management of Acute Malnutrition (IMAM) protocol for timely weight gain. There is limited information on the determinants of consumption of the correct amount of RUTF. This study sought to fill this gap by establishing the associations between the caregivers' and the child's characteristics and the amount of RUTF the child ate within a 24‐h recall period. We used a cross‐sectional study design and interviewed 200 caregivers of children 6–23 months of age admitted in four OTP centers in Nairobi Kenya. We used a researcher‐administered questionnaire to collect information from the caregivers. Seventy‐three percent of the children ate the recommended amount of RUTF. A smaller proportion (54.4%) of younger children (6–11 months of age) ate the recommended amount of RUTF compared to older children (12–17 months old and 18–23 months old at 89.1% and 82.8%, respectively). The predictors of consumption of the correct amount of RUTF were child's birth order—firstborn (AOR 29.92; 95% CI: 5.67–157.93) and children's age; 12–17 months old (AOR 5.19; 95% CI: 2.18–12.36) and 18–23 months (AOR 6.19 95% CI: 2.62), indicating that firstborn and older children were more likely to consume the correct amounts of RUTF. Caregivers' knowledge and correct practices in feeding a child with RUTF also predicted the consumption of the correct amount of RUTF. In conclusion, maternal and child characteristics are determinants of the consumption of the correct amount of RUTF by children in OTP.

## Graphical abstract

Two‐hundred caregivers were assessed, where 73% of the children consumed the recommended amount of RUTF. A smaller proportion (54.4%) of children aged 6–11 months consumed the recommended amount of RUTF compared to older children. Predictors of amount of RUTF intake were child's birth order—firstborn (AOR 29.92; 95% CI: 5.67–157.93) and children's age; 12–17 months (AOR 5.19; 95% CI: 2.18–12.36) and 18–23 months (AOR 6.19 95% CI: 2.62) and caregivers' knowledge and practices in feeding a child with RUTF.

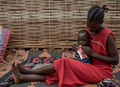

## 1 INTRODUCTION

Children 6–23 months of age are within the critical window of opportunity for foundational growth and development; hence, they need good nutrition to achieve this requirement (Ministry of Health, 2018a; World Health Organisation, 2020). During this stage, suboptimal nutrition greatly compromises childhood developmental milestones and may cause permanent nutritional and health complications in the future (Ireri et al., 2020; World Health Organisation, 2013). Children born in urban informal settlements in Kenya are often at risk of suboptimal breastfeeding and complementary feeding practices, thus increasing their risk of mortality and delayed psychomotor development (Ireri et al., 2020; Kimani‐Murage et al., 2011). Additionally, compromised sanitation within the settlements exposes infants and young children further to higher risks of infectious diseases, thus worsening the vicious cycle of malnutrition (Kimani‐Murage et al., 2011). Limited resources and unsustainable household income are prevailing circumstances in these settings and reduce the caregivers' food purchasing power, thus limiting their ability to diversify the child's diet (Ministry of Health, 2020; Mutisya et al., 2015).

Worldwide, 47 million children 6–59 months of age are wasted, of which 14.3 million are severely wasted. Of the 47 million, 25% are from Sub‐Saharan Africa (World Health Organisation, 2020). In Kenya, 369,379 children 6–59 years of age are acutely malnourished, including 78,908 children with severe acute malnutrition (SAM) (Ministry of Health, 2020). In Nairobi, 44,327 children 6–59 months of age are wasted, including 11,904 children with severe wasting, which is 59% of total SAM prevalence in Kenyan urban centers (Ministry of Health, 2020). The global acute malnutrition (GAM) rate (SAM rate + Moderate Acute Malnutrition [MAM] rate) for Nairobi was 3.9% in July 2019 (Concern Worldwide, 2020).

The Outpatient Therapeutic Program (OTP) for the treatment of severe acute malnutrition for children 6–59 months of age was operationalized in 2008 in Kenya (Wambani, 2012) with 80 government health facilities having functional OTP centers in Nairobi (Concern Worldwide, 2014). In line with the World Health Organization (WHO) guidelines, the children are treated with a specified number of take‐home Ready‐to‐Use Therapeutic Food (RUTF) sachets based on body weight (to attain 200 kcal/kg body weight/day for 7 days), along with specialized routine medication (World Health Organisation, 2020). This RUTF is an energy‐dense, micronutrient‐rich fortified paste, mixed with peanut paste, oil, sugar, and dry milk products, procedurally used to manage and rehabilitate children with severe acute malnutrition (SAM). The children are reviewed weekly at the health facility where they receive the weekly rations of RUTF in proportion to their body weight and are followed up at the community level until they recover from severe acute malnutrition. The OTP centers are located within the residence of the children to reach as many children with SAM as possible (Wambani, 2012).

Several studies have reaffirmed the efficacy of the RUTF in the successful treatment of severe acute malnutrition (Gera, 2010; Okuku et al., 2012; Schoonees et al., 2013). The general findings from these studies showed significant efficacy, safety, and effectiveness of ready‐to‐use‐therapeutic food (RUTF) in treating uncomplicated severe acute malnutrition, with the child achieving a weight mean difference (WMD) ranging from 1.12 g to 3.4 g kg^−1^ day^−1^ if the recommended amount of RUTF is consumed. None of the studies focused on the actual intake of the RUTF by the affected children and the factors determining achievement of adequate intake of RUTF. Consumption of an inadequate amount of RUTF by children with SAM translates to less nutrient intake per day, hence delaying recovery and lengthening the treatment period (Okuku et al., 2012). A study in Burkina Faso showed that reduced RUTF dose slows down the height gain velocity of the children, hence compromising optimal children growth (Kangas et al., 2019). The aim of this study was therefore to establish the key determinants of daily adequate intake of RUTF among the children admitted to OTP in four OTP centers in Kamukunji sub‐county, Nairobi County, Kenya.

## 2 MATERIALS AND METHODS

### 2.1 Study design and study variables

We used a cross‐sectional analytical design with quantitative approaches to data collection, analysis, and presentation. This design enabled the studying of multiple outcome and exposure variables in a representative sample, at one point in time (Sedgwick, 2014). The dependent variable was adequate intake of RUTF. The independent variables were caregivers' socioeconomic characteristics, such as occupation and education level and demographic characteristics, such as caregiver's marital status, age, and parity, demographic characteristics of the child (age, sex, and birth order), and caregivers' knowledge and practices for feeding a child with SAM.

### 2.2 Study site

The study was conducted in four Out‐Patient Therapeutic Programme sites in Kamukunji Sub‐County, Nairobi City County: Bahati, Majengo, Eastleigh, and Biafra health centers. Each of these centers had nutritionists, nurses, and other cadres of health workers. The health facilities had operational OTP centers, integrated with the child welfare clinics within the same facility. Nutrition services offered in the OTP are integrated within the mother and child health program with strong linkage with the outpatient pediatric treatment unit, in keeping with the Kenya Integrated Management of Acute Malnutrition (IMAM) guidelines (Ministry of Medical Services & Ministry of Public Health and Sanitation, 2009; Wambani, 2012). The selected health facilities where this study was conducted are all public health facilities that get technical support from Concern Worldwide, an International Non‐Governmental Organization working in partnership with the Ministry of Health. The NGO provided support in the form of capacity building, commodity supply for the RUTF, whereas the government provided support in the form of supply of essential drugs and support supervision. The support provided by the government and the NGO ensures that the capacity of OTP staff and the community health volunteers are built and that essential nutrition commodities and treatment drugs are consistently available to provide effective and timely service delivery in the management of children with severe acute malnutrition. The caregivers do not pay for this service. In this study, we targeted caregivers with children aged 6–23 months admitted into the OTP centers in Kamukunji sub‐county, for the treatment of severe acute malnutrition.

The four study locations have one thing in common, in that the majority of beneficiaries to which they provide essential health services are communities residing in Nairobi's informal settlements. These include: Kiambiu, Biafra, City Carton, Bahati, and Pumwani‐Majengo informal settlements. Lack of access to safe, adequate and running water, inadequate sanitation facilities, inconsistent electricity supply, use of unsafe cooking fuel (paraffin, charcoal, and firewood), insufficient education support, access challenges to appropriate health and nutrition services, lack of enough space for shelter, and insufficient finances are the most common challenges in these settlements (Kimani‐Murage et al., 2011; Otieno, 2014). Most of the caregivers are involved in casual labor, petty trading, small‐scale manufacturing (*Jua Kali*), and illicit activities, for example, brewing unhygienic liquor. About half (51%) of residents in these informal settlements live in overcrowded conditions (Otieno, 2014). There is a widespread inappropriate infant and young child feeding (IYCF) and high rates of food security in the informal settlements (Ireri et al., 2020; Kimani‐Murage et al., 2011; Macharia et al., 2018; Simiyu et al., 2019), thus children are more likely to be exposed to suboptimal breastfeeding and complementary feeding practices which are major determinants of acute malnutrition.

### 2.3 Sampling procedure

We purposively sampled the four OTP centers (Biafra, Majengo, Eastleigh, and Bahati) because it was necessary to target specifically the caregivers with children admitted to OTP with severe acute malnutrition, and it is these centers that have OTP services. We also considered homogeneity in living conditions (urban informal settlements) for the purposive sampling as all the four health facilities are situated in a strategic location where they serve the majority of families residing in the urban informal settlements. We recruited all the 200 caregivers and their children 6–23 months of age in the four OTP centers who were enrolled into the program at the time of the study and who met the inclusion criteria, upon obtaining their voluntary, informed consent (Martinez‐Mesa et al., 2016).

### 2.4 Inclusion and exclusion criteria

Caregivers with children 6–23 months of age admitted to outpatient therapeutic program in the four specified health facilities in Kamukunji Sub‐county, presenting with severe acute malnutrition (SAM) without complications, having passed the appetite test. Children with edema grade 1 (+) without any medical complications were included in the study. In all cases, voluntary informed consent was obtained from the caregiver. We had planned that should any child sampled for the study develop medical complications (heart disease, Spina Bifida, vomiting, and presence of grade 2 or grade 3 nutritional edema [++, +++]) during the time of the study and be referred to the stabilization/inpatient center before the caregiver was interviewed, the same would be excluded from the sample. However, there were no such cases and therefore we retained the 200 sample of caregivers and their children aged 6–23 months old.

### 2.5 Data collection instruments and data collection procedure

Data collection was conducted by three research assistants with a Diploma level of qualification in Nutrition under the supervision of the researchers. The research assistants were trained by the researchers before data collection. The caregivers were interviewed face to face, using a researcher‐administered questionnaire. The questionnaire was content‐validated and pre‐tested with about 10 mothers who were not included in the main study. During this pre‐test, the test–retest method was used to ensure reliability, by conducting two interviews 7 days apart (Batterham, 2011; Kothari & Garg, 2014). The questionnaire yielded a correlation coefficient of 0.7 using the Cronbach's Correlation formula, which is acceptable (Kothari & Garg, 2014). Some adjustments were made to the questionnaire after pre‐test.

The questionnaire had questions on caregiver's knowledge and practices, such as breastfeeding and the amount of RUTF the child ate in the last 24 h, giving water to the child to drink, safe storage of RUTF, washing hands before feeding the child, and not giving any other complementary food when the child was on RUTF therapy. The interviews were conducted at the OTPs on the days the caregivers and their children had scheduled weekly clinic appointments at the health facilities.

The questions assessing the feeding practices among the caregivers were based on the Kenya Ministry of Health Integrated Management of Acute Malnutrition (IMAM) protocols (Ministry of Medical Services & Ministry of Public Health and Sanitation, 2009) for feeding a child with severe acute malnutrition, which are based on the WHO guidelines. These guidelines stipulate that children admitted to OTP should be on RUTF therapy and are only permitted to breastfeed, according to IMAM protocols (Ministry of Medical Services & Ministry of Public Health and Sanitation, 2009).

Adequate intake of RUTF for children with SAM was determined by calculating the proportion of children 6–23 months of age, who consumed 200 kcal of RUTF per kilogram of body weight the day before the survey. The assessment considered the content of the RUTF given to the children with SAM as an exclusive meal because it contains all the required energy and micronutrients to meet the nutritional needs of children with severe acute malnutrition. Each sachet of RUTF supplies 500 kcal (Ministry of Medical Services & Ministry of Public Health and Sanitation, 2009); hence, the daily rations based on the weight of the child are further calculated in terms of the number of sachets, for the sake of easy understanding and administration by the caregiver. Adequate dietary intake, therefore, refers to the consumption of an adequate amount of RUTF by the child with SAM, according to IMAM protocol. During the interview, the weight of the child was taken from what was recorded in the OTP ration card during the last appointment and recorded by the researcher, and the mother was asked to state the amount of RUTF (in form of sachets) the child ate on the day prior to the survey. The information on the weight and the number of RUFT sachets consumed was recorded in the researcher‐administered questionnaire, and from this an appropriate response was recorded as to whether the child consumed adequate amount of RUTF or not.

### 2.6 Data analysis

Data analysis was conducted using the SPSS software (version 22). Descriptive statistics (frequencies, means, medians, standard deviations, and percentages) were used to describe the caregivers and child demographic characteristics and maternal knowledge and practices on feeding a child with SAM. The Caregivers' knowledge on feeding a child with SAM was based on eight knowledge items out of which the mean knowledge score was calculated. One point was awarded for each correct answers resulting in a total score of 8, whereas zero was awarded for incorrect answers, and thus the least score was zero. Chi‐square test was used to establish associations between categorical variables. Multiple logistic regression was performed to establish the determinants of consumption of the correct amount of RUTF in the last 24 h among the children with SAM. A *p*‐value of <.05 was used as a criterion for statistical significance.

## 3 RESULTS

### 3.1 Child and caregiver's demographic and socio‐economic characteristics and their relationship with consumption of adequate amount of RUTF

A total of 200 caregivers with children aged 6–23 months consented and participated in the study, resulting in a 100% response rate. There was a relatively equal representation of both sexes of the children, 52% being female while 48% were male children. Most of the children with SAM (40.5%) were in the age bracket of 6–11 months followed by children 18–23 months of age (32%). Most (94%) of the caregivers were females, the majority being young mothers between 21 and 30 years of age. The mean age of the caregivers was 27.1 ± 7.6 years. Almost three‐quarters (73.0%) of the caregivers were married. A majority of the caregivers (71%) were either first‐ or second‐time mothers with one or two children. About half of the caregivers (47.5%) had a primary level of education, while 21% had completed secondary level of education. The main occupation for the caregivers was being a housewife (44.5%), followed by casual labor (29.5%). In terms of monthly income, 40.5% of the caregivers earned between 0 and 5000 Kenya shilling (1US$ = Ksh.110.86 as of 25th October, 2021), 33.5% earned between 5001 and 10,000 Kenya shillings. In most households (70%), fathers were responsible for food provision. Most (97.5%) of the houses were iron‐sheet roofed and iron‐sheet‐walled (71.0%). In terms of flooring, 58.5% of the houses were concrete‐floored while the remaining 41.0% were earthen floors. Majority of the caregiver's (67.0%) used paraffin as a source of fuel, while 26.5% used charcoal. About half of the caregivers (50.5%) obtained water for domestic use from communal tap/pump while 48.0% obtained water piped from a tap outside their houses.

Chi‐square test was conducted to show the association between the child's and caregiver's demographic and socio‐economic characteristics, with adequate consumption of RUTF. The consumption of the adequate amount of RUTF per day was significantly associated with children's birth order, child's morbidity status, the caregivers' occupation, and educational level. A child who was not sick the day before the interview was more likely to consume adequate amount of RUTF (χ^2^ = 5.816; *p* = .025) than the child who was ill. Children who were firstborn in the family were more likely to consume an adequate amount of RUTF (χ^2^ = 6.872, *p* = .032). A greater proportion of children whose caregivers were housewives and casual laborers consumed the required amount of RUTF compared to those whose caregivers were engaged in formal employment or engaged in business (χ^2^ = 8.78, *p* = .003). The children of caregivers with upper or secondary level education were significantly more likely to consume the correct amount of RUFT (χ^2^ = 5.097, *p* = <.001) than those whose caregivers had a lower level of education.

Mann–Whitney (*U*‐test) was conducted to show the significant difference between child's and caregiver's characteristics and the consumption of adequate amount of RUTF. A significant difference was observed between the age of the child and consumption of adequate amount of RUTF. Among the children who consumed the adequate amount, majority of them were significantly older compared to those who did not consume the correct amount (Mann–Whitney *Z*‐value = −5.541, *p* = <.001). Another significant difference was observed between the caregiver's age and consumption of adequate amount of RUTF. Majority of the children who consumed the correct amount of RUTF were from significantly older caregivers compared those who did not consume the correct amount (Mann–Whitney *Z*‐value = −1.895, *p* = <.047). Table 1 below summarizes child's and caregiver's characteristics and their relationships with consumption of adequate amount of RUTF by the child with severe acute malnutrition (Table 1).

**TABLE 1:** Child's and caregiver's characteristics and their relationship with consumption of adequate amount of RUTF per day

Variables	Children Consumed the Correct amount of RUTF?							
	Yes		No		Sub‐totals		Chi‐square/Fisher's exact	*p* ‐ value
					*N* = 200			
	*n*	%	*n*	%	*n*	%	(χ2 ) value	
Caregiver's marital status								
Married	108	54	38	19	146	73	6.872	.063
Separated	19	9.5	11	5.5	30	15		
Widowed	2	1	3	1.5	5	2.5		
Single never married	17	8.5	2	1	19	9.5		
Total	146		54		200			
Child's birth order								
1st	73	36.5	29	14.5	102	51	6.995	.032^a^
2nd	58	29	13	6.5	71	35.5		
3rd	15	7.5	12	6	27	13.5		
Total	146		54		200			
Child's age in completed months								
6–11 months	44	22	37	18.5	81	40.5		.001^a^
12–17 months	49	24.5	6	3	55	27.5		
18–23 months	53	26.5	11	5.5	64	32		
Total	146		54		200			
Caregiver's occupation								
Business	18	9	7	3.5	25	12.5	8.78	.003^a^
Casual laborer	56	28	32	16	88	44		
Formal employment	13	6.5	1	0.5	14	7		
Housewife	59	29.5	14	7	73	36.5		
Total	146		54		200			
Caregiver's education level								
No education	4	2	3	1.5	7	3.5	25.097	.001^a^
Lower primary	8	4	14	7	22	11		
Upper primary	68	34	27	13.5	95	47.5		
Secondary not complete	32	16	8	4	40	20		
Secondary complete	34	17	2	1	36	18		
Total	146		54		200			

	Median (IQR)	Median (IQR)	Mann–Whitney *U* value	
Caregiver's age	26 (10)	24 (7)	1.985	.047^a^
Child's Age in Months	14 (9)	7 (6)	5.541	.001^a^

^a^ Significant at 95% confidence interval, *p* value <.05.

### 3.2 Caregiver's knowledge on feeding a child with severe acute malnutrition and their association with Consumption of adequate amount of RUTF

A majority (88%) of the caregivers could explain that ready‐to‐use therapeutic food (RUTF) was the food used to treat children with SAM. The majority (87.3%) knew the number of RUTF sachets their children should be given per day based on their weight (200 kcal kg^−1^ day^−1^) as per the instructions given to them by the health workers. More than three‐quarters of the caregivers (77.6%) knew that plenty of drinking water should be given to the child immediately after taking the RUTF. Most of the caregivers (79.7%) knew that appropriate administration of RUTF with frequent breastfeeding alone without any additional solid or liquid food was enough to help the child regain normal weight and recover from severe malnutrition. All the mothers knew that a child should continue breastfeeding while undergoing treatment for severe acute malnutrition.

On the whole, caregivers' knowledge was statistically significantly associated with the consumption of the correct amount of RUTF. Significant associations were observed for the children of caregivers who were knowledgeable on the following: the need to wash hands before handling and giving RUFT (χ^2^ = 8.514; *p* = .05); the correct amount of RUFT that the child should consume per day (χ^2^ = 15.70; *p* < .001); a child should be given water to drink immediately after feeding on RUTF (χ^2^ = 9.744; *p* = .002); and that RUTF should be kept in a clean and dry place to ensure safety were likely to consume the correct amount of RUTF (χ^2^ = 24.366; *p* < .03) shown in Table 2. The other aspects of caregiver knowledge; the importance of giving the child the correct amount of RUTF per day and RUFT helps the child to regain weight and good health did not have a significant association with the consumption of the correct amount of RUTF (Table 2).

**TABLE 2:** Caregivers' knowledge on feeding a child with SAM and its association with consumption of adequate amount of RUTF

Caregivers' Knowledge	Children Consumed Adequate amount of RUTF/day?								
	Yes		No		Sub‐totals			Chi‐square/Fisher's exact	*p*‐value
	*n*	%	*n*	%	*n*		%		
Knew that RUFT help child to regain weight and good health									
Yes	107	53.5	52	26	159	79.5		2.45	.148
No	39	19.5	2	1	41	20.5			
Total	146		54		200				
Knew they need to wash hands before handling and giving RUFT									
Yes	136	68	32	16	168	84		8.514	.005^a^
No	10	5	22	11	32	16			
Total	146		54		200				
Knew the correct amount of RUFT to be given per day									
Yes	142	71	33	16.5	175	87.5		15.79	<.001^a^
No	4	2	21	10.5	25	12.5			
Total	146		54		200				
Knew that no food should be given to the child when on RUTF									
Yes	136	68	52	26	188	94		3.426	.064
No	10	5	2	1	12	6			
Total	146		54		200				
Knew that a child should be given drinking water immediately after feeding on RUTF									
Yes	129	64.5	26	13	155	77.5		9.744	.002^a^
No	17	8.5	28	14	45	22.5			
Total	146		54		200				
Knew that RUTF should be kept in a clean and dry place to ensure safety for consumption									
Yes	115	57.5	8	4	123	61.5		24.366	.0003^a^
No	31	15.5	46	23	77	27			
Total	146		54		200				

^a^ Significant at 95% confidence interval, *p* value <.05.

### 3.3 Caregivers' feeding practices for a child with SAM and their association with consumption of adequate amount of RUTF

Caregivers' practices on RUTF were determined based on a 24‐h recall and the findings are presented in Table 3. Overall, the majority of the caregivers' practices were appropriate for the feeding of children with SAM. A large majority 97.5% and 96.5% gave the child RUTF from the sachet directly and water to drink immediately after consuming RUTF, respectively. Over two‐thirds (69.5%) and about three‐quarters (76.5%) washed their hands and those of the child before handling and giving the child RUTF sachets and continued breastfeeding the child while on RUTF, respectively. The findings of this study show that 73% of the children consumed the correct quantity of RUTF that is 200 kcal/kg on the day before the survey. The findings show that a lower percentage of younger children 54.3% consumed the correct amount of RUFT compared to the older ones (over 80%).

**TABLE 3:** Caregivers' feeding practices and their association with consumption of adequate amount of RUTF

Variables	Children Consumed Adequate amount of RUTF/day?							
Caregivers' Feeding Practices	Yes		No		Sub‐totals		Chi‐square/Fisher's exact	*p*‐value
	*n*	%	*n*	%	*n*	%		
Caregivers washed their hands and those of the child before handling and giving the RUTF to the child								
Yes	111	55.5	8	4	119	69.5	10.803	.003^a^
No	35	17.5	46	23	81	30.5		
Total	146		54		200			
Caregiver gave the child RUTF direct from the sachets								
Yes	142	71	53	26.5	195	97.5	2.833	.092
No	4	2	1	0.5	5	2.5		
Total	146		54		200			
Caregiver continued breastfeeding their child during treatment for SAM								
Yes	146	73	0	0	153	76.5	233.304	.004^a^
No	0	0	54	27	47	23.5		
Total	146		54		200			
Caregiver gave the child water to drink immediately after feeding him with the RUTF								
Yes	144	72	49	24.5	193	96.5	9.744	.002^a^
No	2	1	5	2.5	7	3.5		
Total	146		54		200			
Caregiver consistently took the child to the health facility weekly for review by the health worker								
Yes	139	69.5	52	26	191	95.5	0.009	.998
No	7	3.5	2	1	9	4.5		
Total	146		54		200			

^a^ Significant at 95% confidence interval, *p* value <.05.

Relationship between caregivers' feeding practices for a child with SAM and consumption of the adequate amount of RUTF was assessed using Chi‐square or Fisher's exact test. The following practices were statistically significantly associated with the consumption of correct amount of RUTF: washing of hands before handling and giving the RUTF sachets to the child (χ^2^ = 10.803; *p* = <.003); child was breastfed the previous day (χ^2^ = 223.203; *p* = <.001); and child drank water during and after feeding on RUTF (χ^2^ = 9.744; *p* = .002) as shown in Table 3 below.

### 3.4 The predictors of consumption of the correct amount of RUTF

Multiple logistic regression was performed to establish the predictors of consumption of the correct amount of RUTF in the last 24 h among the children. The model contained 13 independent variables which had an association with the consumption of the correct amount of RUTF in univariate analysis. After controlling for caregiver's age, since most of the caregivers were young (21–30 years of age), the full model containing all predictors was statistically significant, χ^2^ (13, *n* = 200) = 233.304, *p*‐value <.01, indicating that the model could distinguish between children who consumed the correct amount of RUTF and those who did not.

Children who were firstborn (first birth order) were 30 times more likely to consume the correct amount of RUTF while second birth order children were 13 times more likely to consume the correct amount of RUTF in the last 24 h (AOR; 29.916; 95% CI: 5.667–157.931 and AOR 12.565; 95% CI:2.799–53.176), respectively, compared to those who were thirdborns. Older children age groups 12–17 months of age were five times more likely to consume the correct amount of RUTF while those 18–23 months of age were six times more likely to consume the correct amount of RUTF in the last 24 h compared to younger children (age group 6–11 months; AOR; 5.192; 95% CI: 2.181–12.360 and AOR; 6.186; 95% CI:2.618–14.615). Children whose caregivers had knowledge on the need to wash hands before giving RUTF were 8.5 times more likely to consume the correct amount of RUTF than those whose caregivers did not have this knowledge (AOR; 8.514; 95% CI:8.44–8.77). Children whose caregivers knew the importance of giving water to drink after RUTF feeding were 10 times more likely to consume the correct amount of RUTF compared to those whose mothers did not have this knowledge (AOR; 9.744; 95% CI:8.66–9.997). Similarly, caregivers who knew the importance of storing RUTF in a cool dry place were 24 times more likely to feed their children the correct amount of RUTF compared to those who did not know safe storage (AOR; 24.37; 95% CI:23.44–26.55). In terms of caregiver practice, children whose caregivers washed their hands before feeding them with RUTF were 11 times more likely to consume the correct amount of RUTF than those whose caregivers did not wash their hands (AOR; 10.8; 95% CI:9.97–11.25). Children who were breastfed the previous day were 20 times more likely to consume the correct amount of RUTF compared to those who were not breastfed (AOR; 20.0; 95% CI:19.12–21.33). Similarly, children who drank water while feeding on RUTF were 10 times more likely to consume the correct amount of RUTF compared to those who were not given water to drink during feeding (AOR; 9.74; 95% CI:9.44–9.87). Children who were not sick the previous day were five times more likely to consume the correct amount of RUTF compared to those who suffered illness the previous day (AOR; 5.82; 95% CI:4.11–6.13; Table 4).

**TABLE 4:** The predictors of consumption of the correct amount of RUTF

Variables		95% CI for AOR	
	AOR	Lower	Upper
Caregiver's and child's demographic characteristics:			
Child's birth order			
1st	29.916^a^	5.667	157.931
2nd	12.565^a^	2.969	53.176
3rd	REF		
Caregiver's occupation			
Business	0.501	0.128	1.962
Casual laborer	0.362	0.138	0.947
Formal employment	0.71	0.048	10.516
Housewife	REF		
Caregiver's education level			
No education	0.108	0.01	1.219
Lower primary	0.029	0.004	0.228
Upper primary	0.184	0.032	1.059
Secondary not complete	0.385	0.057	2.594
Secondary complete	REF		
Caregiver's age	1.048	0.978	1.124
Child age group in months			
6–11 months	REF	.	.
12–17 months	5.192^a^	2.181	12.36
18–23 months	6.186^a^	2.618	14.615
Caregivers' knowledge			
Need to wash hands before handling and giving RUFT			
Yes	8.514^a^	8.44	8.77
No	REF		
Food should be given to the child when on RUTF therapy			
Yes	3.42	0.45	3.78
No	REF		
Child should be given drinking water immediately after feeding on RUTF			
Yes	9.744^a^	8.66	9.997
No	REF		
RUTF should be kept in a clean and dry place to ensure safety for consumption			
Yes	24.37^a^	23.44	26.55
No	REF		
Caregiver practices on handling of RUTF			
Washed hands before handling and giving the RUTF sachets to the child			
Yes	10.8^a^	9.97	11.25
No	REF		
Child breastfed the previous day			
Yes	20^a^	19.12	21.33
No	REF		
Child was given water after feeding on RUTF			
Yes	9.74^a^	9.44	9.87
No	REF		
Child's morbidity status:			
Child sick in the last 14 days			
Yes	REF		
No	5.82^a^	4.11	6.13

^a^ Significant at 95% CI, *p* value <.05.

## 4 DISCUSSION

Appropriate therapeutic feeding for severe acutely malnourished children is key to timely achievement of nutritional recovery, hence the need to ensure daily and consistent consumption of appropriate amount of RUTF by the child with SAM admitted in Outpatient Therapeutic Programmes (OTP) (World Health Organisation, 2013). Lack of or poor adherence to RUTF therapy may lead to failure to attain the desired weight gain by the child within the recommended period, thus resulting into delayed milestones, long treatment period, and increased risk of defaulting from the program, (Gera, 2010; Schoonees et al., 2013) thus making the program less effective.

There is scarcity of information on the amounts of RUTF consumed by the children admitted to OTP and the determinants of the consumption of the correct amount of the product. To the best of our knowledge, our study was the first in Kenya to establish the determinants of the amount of RUTF consumption for children in OTP. In our study, consumption of RUTF was measured based on a 24‐h recall as reported by the children's caregivers.

The findings that nearly three‐quarters of the children consumed the correct amount of RUTF show that majority of the children got adequate nutrients for timely nutritional rehabilitation and recovery. However, the highest proportion of children 6–11 months of age failing to consume adequate amount of RUTF may be attributed to the fact that most of the younger children were at their initial stage of introduction to solid foods and may have had difficulty acquainting themselves with the solid RUTF. More in‐depth knowledge on this may be obtained from qualitative investigation to ascertain the reasons for low consumption of RUTF, particularly among the younger children. These findings imply that a larger percentage of younger children may not be getting the adequate amount of nutrients to regain the weight based on the Kenya Ministry of Health Integrated Management of Acute Malnutrition (IMAM) protocols (Ministry of Medical Services & Ministry of Public Health and Sanitation, 2009) and as per the WHO Standard Protocols of 200 kcal/kg/per body weight, hence may be at risk of delayed milestones. This finding agrees with the results of the analysis of length of stay in OTP in Marsabit County Kenya that showed that more than 50% of children admitted in OTP in North Horr Sub‐County stayed in the program for 12 weeks or more longer than the recommended 8 weeks' period (Concern Worldwide, 2019). The long stay may be partly explained by consumption of inadequate amounts of RUTF.

The findings that majority of the firstborn children consumed adequate amounts of RUTF compared to the others, together with the fact that most caregivers were young mothers may imply a greater attention given to these children by the caregivers who many may be first‐time mothers. Also considering that the caregivers were mostly mothers of between 1 and 2 children, it may imply a less likelihood of RUTF sharing among siblings when the affected child is an only child in the household than when there is the presence of younger siblings. This finding is in agreement with the findings of the study conducted in Marsabit County, Kenya (Concern Worldwide, 2019) where among the reasons cited for the longer stay of children in OTP than recommended and high caseloads of nonresponse to treatment in North Horr Sub‐County, was the sharing of RUTF among younger siblings.

In this study, presence of child illness predicted less consumption of RUTF, implying that illness has a negative impact on feeding patterns of children and that much more attention needs to be given to ensure the children obtain prompt treatment, breastfeed more frequently, and are given small quantities of RUTF more frequently to help them achieve adequate energy and nutrient intake during illness (World Health Organisation, 2013). This finding concurs with that of a study conducted in Nairobi informal settlements where inadequate nutrient intake and subsequent wasting among infants and young children were significantly associated with common childhood illnesses, such as cough and diarrhea (De Vita et al., 2019).

The findings of this study indicate that caregivers' high knowledge on various aspects of feeding a child with RUTF was a predictor of the consumption of the correct amount of RUTF. This suggests that with appropriate approaches in equipping caregivers with correct knowledge on appropriate therapeutic feeding practices for their children, they are empowered to make informed decisions to translate the knowledge gained into correct and appropriate feeding practices. The finding concurs with findings of a study in health facilities in Ghana (Bimpong et al., 2020) that showed that high knowledge among caregivers in child feeding recommendations positively determined receipt of minimum adequate diet by the children. This finding, however, differs from those reported from a study in Somalia (Gulled et al., 2016) which showed that despite the high knowledge of the participants on Infant and Young Child Feeding (IYCF), a large proportion of mothers/caregivers had poor practice on proper IYCF leading to high rate of suboptimal feeding practices. Important to note is that these two studies were conducted in the context of general complementary feeding and not in the context of feeding a child with SAM.

Appropriate practices in the handling of the RUTF in the feeding of children in OTP also predicted consumption of the correct amount of RUTF. These findings demonstrate that compliance with the correct practices enhances the consumption of adequate RUTF by the child. The findings further imply that despite having a low level of education, the caregivers were able to understand the guidance given them by the OTP staff and community health volunteers on feeding a child with SAM. This finding demonstrates that with an appropriate approach, barriers of low level of education, language, unfavorable attitudes, or cultural practices can be overcome to help caregivers adopt appropriate feeding practices for a child with SAM. This finding concurs with those reported in a Knowledge Attitudes and Practices (KAP) survey in Samburu (Ministry of Health, 2018b), where despite the caregivers having a low level of education, the majority of caregivers were able to practice exclusive breastfeeding guided by local community health workers.

The findings of this study have demonstrated that despite the free access to RUTF for the treatment of children with severe acute malnutrition, other factors are determining the amount of RUTF that the child consumes. There is a need to investigate the barriers to the consumption of an adequate amount of RUTF by children in OTP and especially by the younger children.

### 4.1 Strengths of the study

This study has provided significant and useful information that contributes to filling the gap currently existing in Kenya on the predictors of adequate consumption of RUTF by children with severe acute malnutrition.

### 4.2 Limitations of the study

This study being a cross‐sectional one, we were not able to capture variability (if any) in the caregiver's practices in the management of a child in OTP using the RUTF therapy over the period the child was admitted in the program. Data on the amount of RUTF consumed depended on the caregivers' recall and thus there was a likelihood of recall bias in the finding. Appropriate attention was given to ensure quality control of data by thorough training of the enumerators, and close supervision of all the study activities.

## 5 CONCLUSIONS

We conclude that both caregivers and child characteristics play a role in determining the amount of RUTF that a child consumes among the study population. Moreover, caregivers' knowledge and practices on feeding a child with SAM are also important predictors of consumption of adequate amount of RUTF. This study has demonstrated that the consumption of the adequate amount of RUTF by severe acutely malnourished children in OTP is not only dependent on access to the product. Despite all the children in the study having access to the correct amount of RUTF, about one‐quarter of the children did not consume the correct amount of the product implying that these children would not gain weight at the expected rate, and therefore stay longer in the program than recommended, thus rendering the program less cost‐effective. There is a need for in‐depth qualitative investigation to establish the barriers to the consumption of the correct amount of RUTF, however. The product is accessible at no cost, and the majority of the caregivers were knowledgeable about the feeding of children in OTP, and their practices on feeding the children were on the whole appropriate. The findings of this study provide valuable information on the research efforts in rehabilitating children with SAM.

## CONFLICT OF INTEREST

The authors declare that they have no conflict of interest.

## Data Availability

The data that support the findings of this study are available from the corresponding author upon reasonable request.
